# Glucobrassicin Metabolites Ameliorate the Development of Portal Hypertension and Cirrhosis in Bile Duct-Ligated Rats

**DOI:** 10.3390/ijms20174161

**Published:** 2019-08-26

**Authors:** Ting Chang, Hsin-Ling Ho, Shao-Jung Hsu, Ching-Chih Chang, Ming-Hung Tsai, Teh-Ia Huo, Hui-Chun Huang, Fa-Yauh Lee, Ming-Chih Hou, Shou-Dong Lee

**Affiliations:** 1 Division of General Medicine, Department of Medicine, Taipei Veterans General Hospital, Taipei 11217, Taiwan; 2 Institute of Pharmacology, National Yang-Ming University School of Medicine, Taipei 11217, Taiwan; 3 Division of Gastroenterology and Hepatology, Department of Medicine, Lotong Poh-Ai Hospital, Yilan 26546, Taiwan; 4 Faculty of Medicine, National Yang-Ming University School of Medicine, Taipei 11217, Taiwan; 5 Division of Gastroenterology and Hepatology, Department of Medicine, Taipei Veterans General Hospital, Taipei 11217, Taiwan; 6 Chang Gung University College of Medicine and Division of Gastroenterology and Hepatology, Chang Gung Memorial Hospital, Taoyuan 33305, Taiwan; 7 Department of Medical Research, Taipei Veterans General Hospital, Taipei 11217, Taiwan; 8 Division of Gastroenterology, Department of Medicine, Cheng Hsin General Hospital, Taipei 11217, Taiwan

**Keywords:** indole-3-carbinol (I3C), 3,3′-diindolymethane (DIM), liver cirrhosis, portosystemic collaterals, angiogenesis

## Abstract

Patients suffering from liver cirrhosis are often complicated with the formation of portosystemic collateral vessels, which is associated with the progression of a splanchnic hyperdynamic circulatory state. Alleviating pathological angiogenesis has thus been proposed to be a feasible treatment strategy. Indole-3-carbinol (C9H9NO, I3C) and 3,3′-diindolymethane (DIM), formed by the breakdown of glucosinolate glucobrassicin, are prevalent in cruciferous vegetables and have anti-angiogenesis properties. We aimed to evaluate their influences on portal hypertension, the severity of mesenteric angiogenesis, and portosystemic collaterals in cirrhosis. Sprague-Dawley rats with common bile duct ligation (CBDL)-induced liver cirrhosis or sham operation (surgical control) were randomly allocated to receive I3C (20 mg/kg/3 day), DIM (5 mg/kg/day) or vehicle for 28 days. The systemic and portal hemodynamics, severity of portosystemic shunting, mesenteric angiogenesis, and mesenteric proangiogenic factors protein expressions were evaluated. Compared to vehicle, both DIM and I3C significantly reduced portal pressure, ameliorated liver fibrosis, and down-regulated mesenteric protein expressions of vascular endothelial growth factor and phosphorylated Akt. DIM significantly down-regulated pErk, and I3C down-regulated NFκB, pIκBα protein expressions, and reduced portosystemic shunting degree. The cruciferous vegetable byproducts I3C and DIM not only exerted a portal hypotensive effect but also ameliorated abnormal angiogenesis and portosystemic collaterals in cirrhotic rats.

## 1. Introduction

Liver cirrhosis with portal hypertension, characterized by increased intrahepatic resistance and splanchnic hyperemia, is considered the end stage of liver diseases. Increased portal inflow to a stiff liver can result in the development of portosystemic collaterals, aiming to divert stagnant blood flow. Gastroesophageal varices are the most serious collaterals clinically, because bleeding from these fragile vessels can be lethal and the treatment efficacy of pharmacological agents is still suboptimal [1]. Recent studies have indicated that angiogenesis participates in the development and aggravation of collaterals and increased portal blood inflow. Vascular endothelial growth factor (VEGF), a potent proangiogenic factor, is overexpressed in splanchnic organs of portal hypertensive animals [2,3]. In addition, splanchnic VEGF receptor 2 (VEGFR2) and endothelial cell marker CD31 are up-regulated in portal hypertensive rats [2]. These findings suggest that anti-angiogenesis may be a new treatment strategy in controlling portal hypertension.

Cruciferous vegetables such as cabbage and cauliflower have been noticed due to their potential in treating cancer and chronic diseases [4,5]. These vegetables contain large amounts of glucobrassicin, an indolylmethyl glucosinolate. When these plants are cut or chewed, a thioglucosidase-mediated autolytic process takes place, generating indole-3-carbinol (I3C). In addition, when being exposed to acid, I3C forms various condensation products [6,7], with 3,3′-diindolymethane (DIM) being the most common. There is approximately 12 mg of DIM in 200 g of broccoli [8,9]. I3C has been shown to prevent the proliferation of various tumor cells, including breast cancer and colon cancer [10]. Furthermore, both I3C and DIM significantly reduced the incidence of 7,12-dimethylbenz(a)anthracene-induced mammary tumors in female rats and benzo(a)pyrene-induced forestomach neoplasia in female mice [11,12].

Of their anti-cancer mechanisms, the anti-angiogenesis effect is remarkable and might be applied to control pathological angiogenesis in cirrhosis. For example, I3C has been shown to decrease the density of CD31-stained prostate tumor microvessels [13], and DIM suppressed the proliferation of cultured human vascular endothelial cells and vascularization of Matrigel plugs in athymic mice [14]. Regarding the mechanism of angiogenesis, p44/p42 mitogen-activated protein kinases (MAPK or Erk1/2), which are activated when VEGF binds VEGFR2, have been linked to VEGF-mediated endothelial cell proliferation [15,16,17,18]. Nitric oxide (NO) also participates in portal hypertension-related angiogenesis [19,20], and its synthesis is stimulated by VEGF [21]. On the other hand, the hypoxia-inducible factor (HIF)-1α regulates VEGF expression and endothelial NO synthase (eNOS), matrix metalloproteinase-9 (MMP-9) also act as pro-angiogenic factors [22,23]. However, whether I3C and/or DIM may mitigate this pathological condition and what the underlying mechanism is have yet to be investigated.

Emerging data show that I3C and DIM exert anti-fibrosis, anti-oxidant, and anti-inflammation effects on the liver through pleiotropic mechanisms [24]. The therapeutic effect of I3C on hepatic fibrosis of rats has been demonstrated [25]. Through de-ubiquitinating the receptor-interacting protein 1, I3C induced apoptosis of hepatic stellate cells then reversed liver fibrosis [26]. In addition, DIM suppressed hepatic stellate cells activation by down-regulating the expression of microRNA-21, and administration of DIM in vivo attenuated liver fibrosis [27].

Considering the anti-angiogenesis and anti-fibrotic effects of I3C and DIM, and the easy access to cruciferous vegetables in daily life, this study investigated the influences of I3C and DIM on the development of portal hypertension, liver fibrosis, and the severity of mesenteric angiogenesis and portosystemic collaterals in rats with common bile duct ligation (CBDL)-induced liver cirrhosis.

## 2. Results

### 2.1. Body Weight, Hemodynamics, Liver, and Kidney Biochemical Parameters

Table 1 shows the hemodynamic parameters and plasma levels of the liver, renal biochemical parameters of the sham, and CBDL-cirrhotic rats treated with I3C, DIM, or vehicle. As compared with the sham-vehicle group analyzed with *t*-test, CBDL-vehicle group had significantly lower body weight. Besides, the lower mean arterial pressure (*p* = 0.017), superior mesenteric arterial resistance (*p* = 0.001), systemic vascular resistance (*p* = 0.001), and higher portal pressure (*p* < 0.001), superior mesenteric artery flow (*p* = 0.032), cardiac index (*p* = 0.008), and stroke volume (*p* < 0.001) in CBDL group indicated that CBDL successfully induced the hemodynamic features of portal hypertension. In CBDL groups analyzed with ANOVA, compared with the vehicle, I3C reduced portal pressure by 15.2% (*p* = 0.033). There were no significant differences in mean arterial pressure, cardiac output, cardiac index, stroke volume, systemic vascular resistance, superior mesenteric artery flow, superior mesenteric artery resistance, and other biochemical markers (all *p* > 0.05). In addition, there was no significant difference in body weight among the three groups. In sham groups analyzed with ANOVA, compared with vehicle, DIM increased cardiac output of 22.8% (*p* = 0.010), cardiac index of 26.4% (*p* = 0.003), stroke volume of 29.8% (*p* = 0.044), and decreased systemic vascular resistance of 20.4% (*p* = 0.003). I3C increased stroke volume of 38.6% (*p* = 0.011). There were no significant differences in plasma levels of liver and kidney biochemical parameters.

### 2.2. Degree of Portosystemic Shunting and Plasma VEGF Concentration

**Figure 1:**
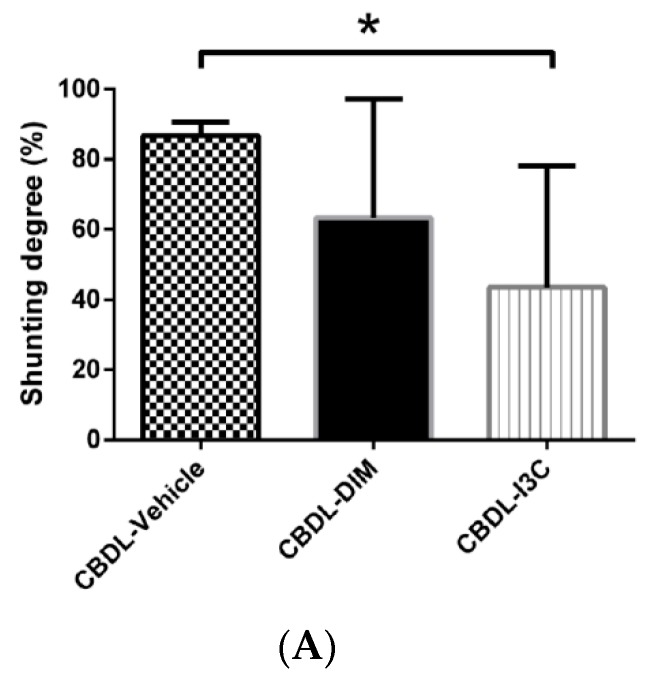

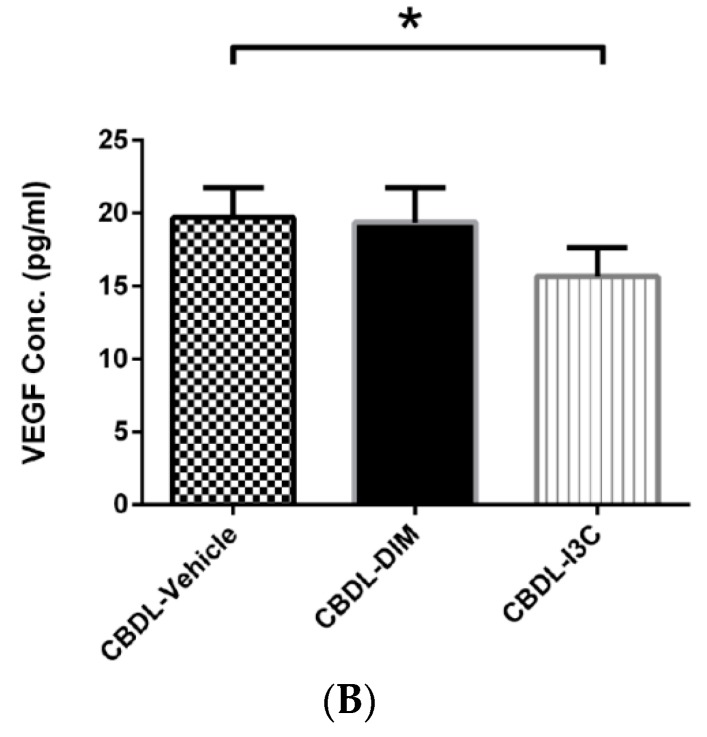
(**A**) The degree of portosystemic shunting of CBDL rats treated with vehicle, DIM (3,3′-diindolymethane), or I3C (Indole-3-carbinol) analyzed with ANOVA. I3C significantly decreased the degree of shunting (* *p* < 0.05, I3C vs. corresponding vehicle group). (**B**) The plasma VEGF (vascular endothelial growth factor) concentrations analyzed with ANOVA. Compared with vehicle, I3C significantly decreased the plasma VEGF concentration (* *p* < 0.05, I3C vs. vehicle).

The degree of portosystemic shunting in CBDL-cirrhotic rats analyzed with ANOVA is depicted in Figure 1A. Compared with vehicle, I3C markedly decreased the degree of shunting by 49.8%. Although DIM tended to reduce the shunting degree, the statistical significance was not reached (CBDL-vehicle (*n* = 4) vs. CBDL-DIM (*n* = 6) vs. CBDL-I3C (*n* = 6): 86.73 ± 3.76% vs. 63.26 ± 33.95% vs. 43.51 ± 34.48%, I3C vs. vehicle, *p* = 0.044; DIM vs. vehicle: *p* = 0.248).

Figure 1B shows the plasma VEGF concentration of CBDL-cirrhotic rats analyzed with ANOVA. Compared with vehicle, I3C significantly decreased the VEGF concentration by 20.4% (CBDL-vehicle (*n* = 5) vs. CBDL-DIM (*n* = 7) vs. CBDL-I3C (*n* = 7): 19.70 ± 2.03 vs. 19.37 ± 2.37 vs. 15.68 ± 1.93, DIM vs. vehicle: *p* > 0.05, I3C vs. vehicle: *p* = 0.005).

### 2.3. Mesenteric Angiogenesis

**Figure 2:**
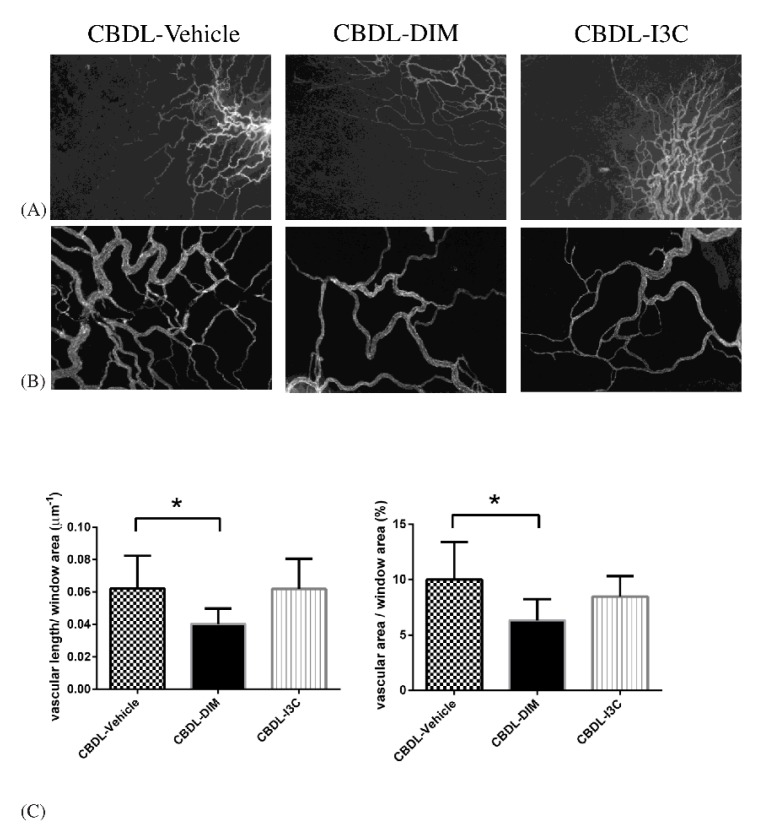
Representative images of CD31-labelled mesenteric window microvascular networks in CBDL rats treated with vehicle, DIM, or I3C: (**A**) The magnification 40×, (**B**) the magnification 100×. (**C**) The vascular density of different experimental groups analyzed with ANOVA under the magnification 100×. DIM markedly decreased the vascular length (* *p* < 0.05, DIM vs. vehicle) and vascular area of mesenteric window (* *p* < 0.05, DIM vs. vehicle).

Figure 2 shows that compared with vehicle, DIM significantly reduced both vascular length per unit window area (μm^−1^) and vascular area per unit window area (%) as analyzed with ANOVA (CBDL-vehicle (*n* = 4) vs. CBDL-DIM (*n* = 7) in vascular length per unit window area (μm^−1^): 0.0622 ± 0.0202 vs. 0.0403 ± 0.0096, *p* = 0.046; in vascular area per unit window area (%): 10.00 ± 3.40 vs. 6.32 ± 1.91, *p* = 0.018) in CBDL-cirrhotic rats. I3C did not significantly influence either parameter (both *p* > 0.05 vs. vehicle).

### 2.4. Angiogenesis-Related Protein Expressions in Mesentery

**Figure 3:**
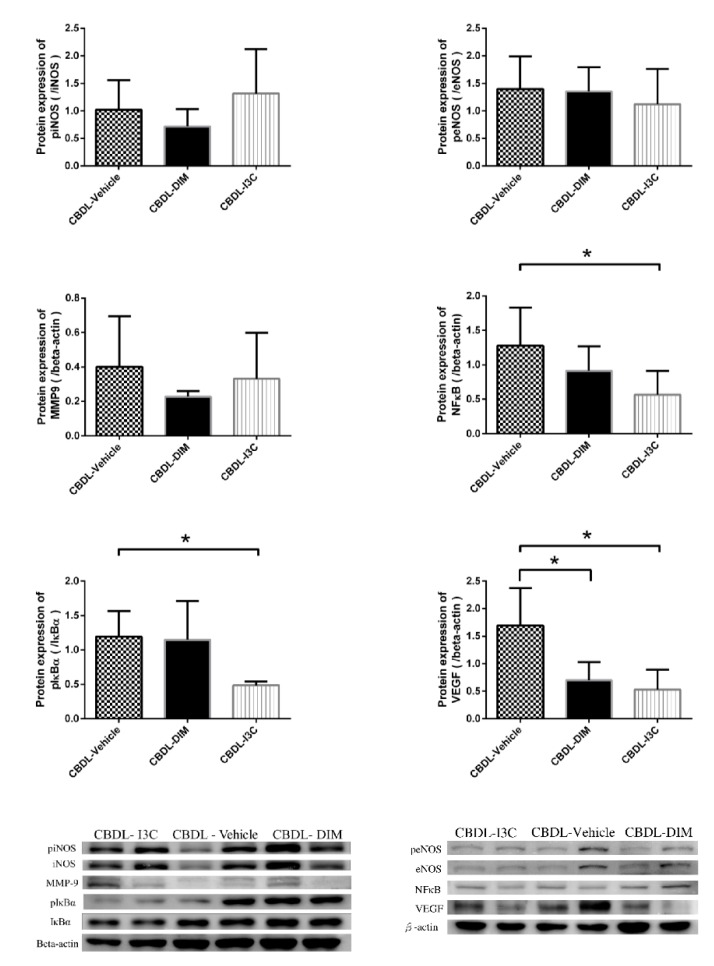
Mesenteric protein expressions of piNOS/iNOS, peNOS/eNOS, MMP-9, NFκB p65, pIκBα/IκBα, VEGF in CBDL rats treated with vehicle, DIM, or I3C analyzed with ANOVA (* *p* < 0.05, DIM- or I3C-treated group vs. vehicle group). The representative images are shown below.

**Figure 4:**
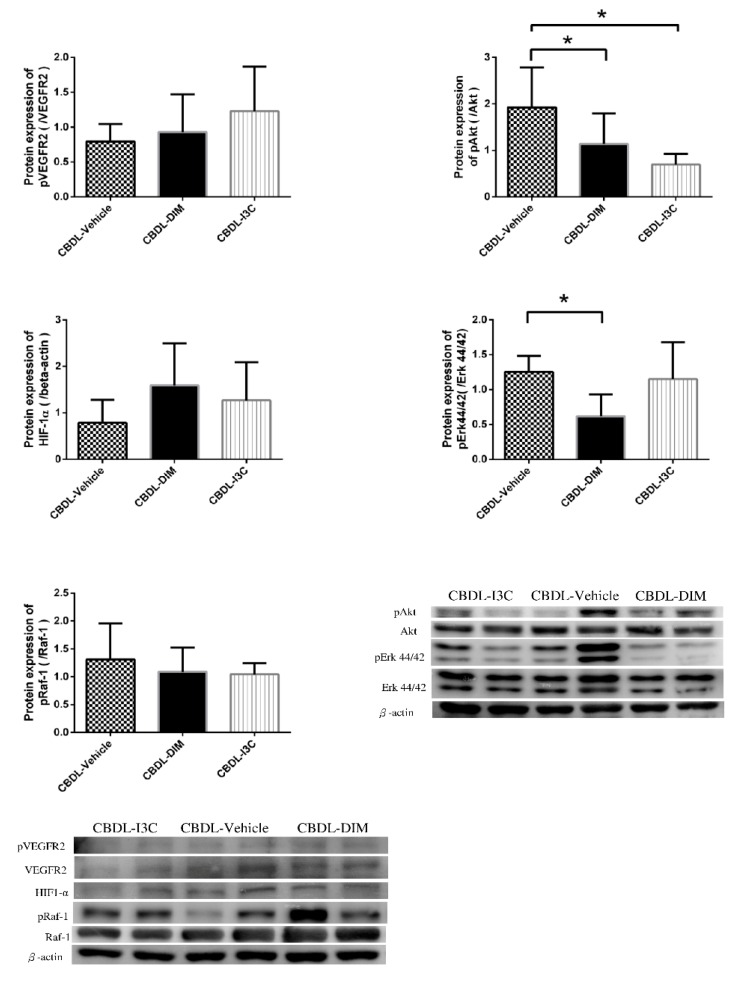
Mesenteric protein expressions of pVEGFR2/VEGFR2, pAkt/Akt, HIF1-α, pErk44/42/Erk44/42, pRaf-1/Raf-1 in CBDL rats treated with vehicle, DIM, or I3C analyzed with ANOVA (* *p* < 0.05, DIM or I3C vs. vehicle). The representative images are shown below.

The mesenteric proangiogenic factor protein expressions of CBDL-cirrhotic rats analyzed with ANOVA are shown in Figure 3 and Figure 4. Compared with vehicle, DIM and I3C significantly down-regulated the expressions of VEGF and pAkt (CBDL-vehicle vs. CBDL-DIM vs. CBDL-I3C (/β-actin): VEGF, 1.69 ± 0.68 vs. 0.70 ± 0.33 vs. 0.53 ± 0.36, *p* = 0.003 and *p* = 0.001 for DIM vs. vehicle and I3C vs. vehicle, respectively; pAkt, 1.92 ± 0.86 vs. 1.14 ± 0.65 vs. 0.69 ± 0.23, *p* = 0.047 and 0.003, respectively). Compared with vehicle, DIM significantly down-regulated pErk (CBDL-vehicle vs. CBDL-DIM: 1.25 ± 0.23 vs. 0.62 ± 0.31, *p* = 0.016). In addition, I3C significantly down-regulated the expressions of NFκB sububit p65 and pIκBα (CBDL-vehicle vs. CBDL-I3C: NFκB p65, 1.28 ± 0.55 vs. 0.57 ± 0.34, *p* = 0.016; pIκBα, 1.19 ± 0.37 vs. 0.49 ± 0.05, *p* = 0.014). Neither DIM nor I3C significantly influenced the protein expressions of pVEGFR2, peNOS, piNOS, pRaf-1, HIF-1α, or MMP-9, as compared with the vehicle (all *p* > 0.05).

### 2.5. Liver Fibrosis

**Figure 5:**
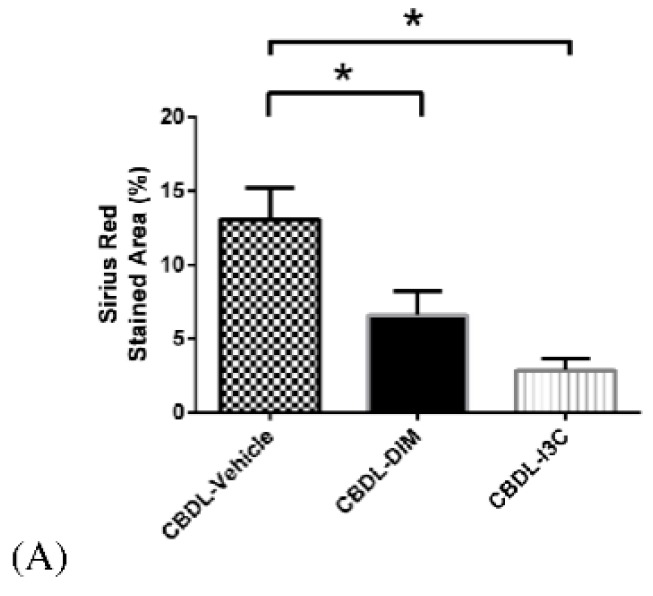

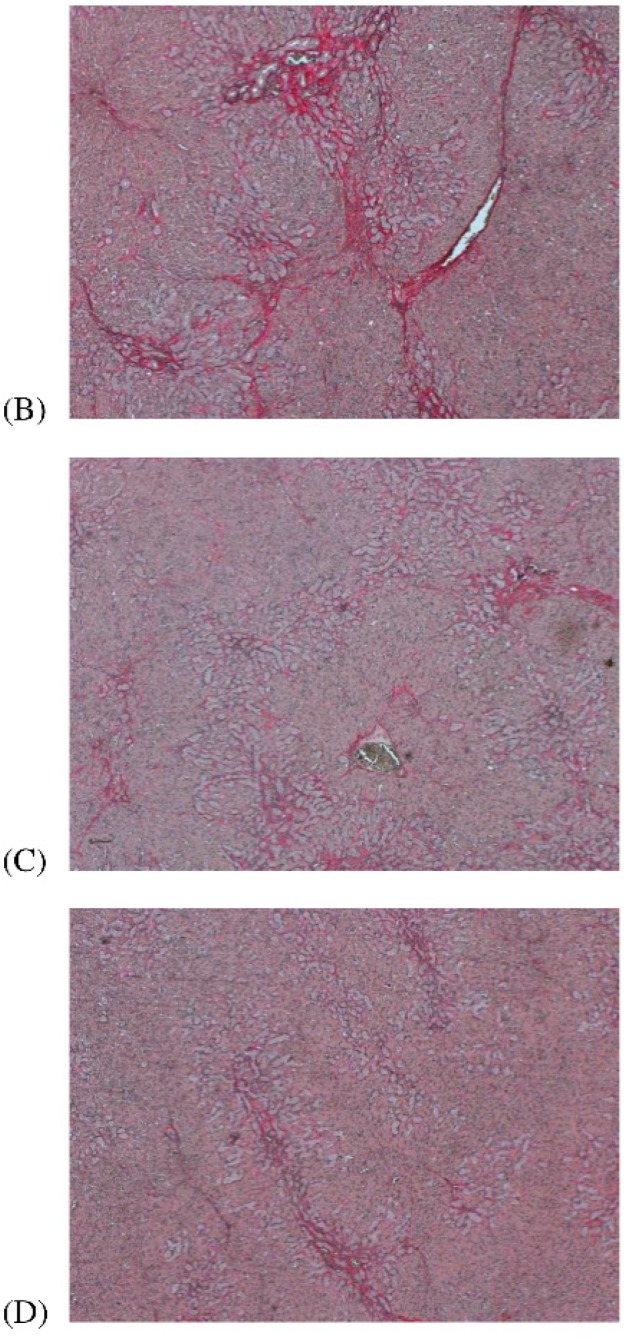
(**A**) The Sirius red-stained area of liver in CBDL rats treated with vehicle, I3C, or DIM analyzed with ANOVA. I3C and DIM significantly decreased the Sirius red-stained area (* *p* < 0.05, DIM or I3C vs. vehicle). The representative images of (**B**) CBDL-vehicle, (**C**) CBDL-DIM, and (**D**) CBDL-I3C groups are shown (in magnification 40×).

Figure 5 shows that, compared with vehicle, DIM and I3C reduced the percentage of Sirius red-stained area by 49.5% and 78.1%, respectively (CBDL-vehicle (*n* = 6) vs. CBDL-DIM (*n* = 7) vs. CBDL-I3C (*n* = 8): 13.09 ± 2.11 vs. 6.61 ± 1.64 vs. 2.87 ± 0.80, DIM vs. vehicle and I3C vs. vehicle: both *p* < 0.05 analyzed with ANOVA).

## 3. Discussion

Both I3C and its dimeric product, DIM, are prevalent in cruciferous vegetables, and they have aroused much attention with regards to their anti-cancer and anti-angiogenesis effects [28,29,30]. In the current study, we found that I3C and/or DIM ameliorated portal hypertension, portosystemic shunting, liver fibrosis, mesenteric vascular density, and down-regulated proangiogenic factors expressions in rats with CBDL-induced liver cirrhosis, although they did not exert exactly the same effects.

The development and progression of portal hypertension is determined by three interactive vascular systems: Hepatic, splanchnic, and portosystemic collateral systems [31]. Each system is composed of structural and functional aspects. Increased intrahepatic resistance derived from fibrosis and exaggerated vasoconstriction is the primary factor determining portal pressure. In addition, splanchnic vasodilation and mesenteric angiogenesis elevate portal inflow and pressure. In collateral vasculature, portosystemic collaterals also develop to divert stagnant portal inflow, with the severity shown by shunting degree [32]. Moreover, the increased collaterals related to angiogenesis enhance splanchnic blood flow and further aggravate portal hypertension. In this study, portal pressure, the dominant pathophysiological marker of liver cirrhosis, was significantly reduced by DIM and I3C. Furthermore, the significant reduction of shunting degree and plasma VEGF level, the main factor involved in angiogenesis in the I3C-treated group suggested that I3C increased collateral vascular resistance by reducing shunting and ameliorating pathological angiogenesis [33]. Furthermore, DIM and I3C did not influence splanchnic hemodynamic derangement, as shown by superior mesenteric artery flow and resistance. Based on these findings, the decrease in intrahepatic resistance may be the main factor driving the portal hypotensive effect. Consistent with this hypothesis, Sirius red staining indicated that both DIM and I3C markedly ameliorated the extent of liver fibrosis, which contributed to reduction of intrahepatic resistance [34]. In addition, we checked the hemodynamic and liver/kidney biochemistry data on corresponding sham groups, showing that DIM or I3C increased cardiac output, cardiac index, stroke volume and decreased systemic vascular resistance, suggesting a beneficial cardiovascular impact of these compounds in normal subjects. Interestingly, this has not been identified in the past. The lack of elevated liver and kidney biochemistry levels also indicate their lack of major organ toxicity.

**Figure 6:**
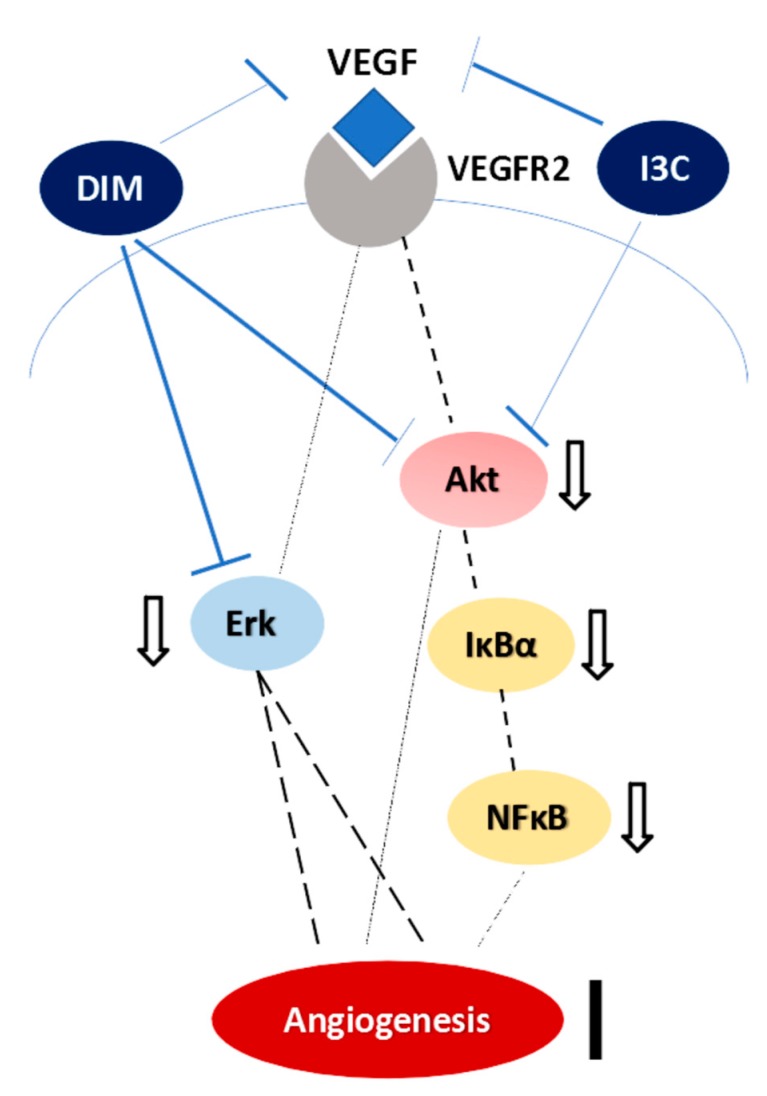
The angiogenesis-related signaling pathways influenced by I3C and DIM in mesentery of rats with CBDL-induced liver cirrhosis. The dashed line denotes the down-regulated signaling transduction, the solid line indicates the blocked angiogenesis, and the t-bar represents the direct inhibitory actions of I3C and DIM on the targets.

Previous investigations have reported that DIM not only inhibits the VEGF-mediated Ras-Raf-Erk1/2 cascade activation but also suppresses NFκB and Akt activation in cancer cells [35,36]. I3C has also been reported to suppress the lipopolysaccharide-induced capillary-like structure formation of endothelial cells and VEGF and NO secretion [37]. In addition, it has been shown to inhibit NFκB and IκBα kinase activation in myeloid and leukemia cells [38]. Consistently, we also found that I3C down-regulated mesenteric VEGF, pAkt, IκBα, and NFκB subunit, p65. Furthermore, DIM significantly suppressed VEGF, pAkt and pErk expressions, suggesting the downregulation of VEGF-mediated ERK signaling pathway (Figure 6). It is worth noting that in CBDL-induced cirrhosis, DIM alleviated the mesenteric angiogenesis more effectively than I3C did.

Although DIM but not I3C significantly ameliorated mesenteric vascular density, I3C, but not DIM, reduced the degree of shunting and plasma VEGF level. This indicates that I3C decreased the whole portosystemic shunting amount while DIM exerted more regional specific effect on mesentery. Nevertheless, these interesting findings suggest that via taking cruciferous vegetables, the source of both I3C and DIM, cirrhotic patients may benefit from both beneficial effects. Indeed, further clinical surveys are warranted.

The previous studies have reported that I3C and DIM own anti-fibrosis, anti-angiogenesis, and anti-inflammation capacities in different tissues [39]. The current study identified that I3C and DIM treatments exerted anti-angiogenesis and anti-fibrosis effects in CBDL rats. However, the anti-inflammation effect was not prominent as evidenced by the similar plasma levels of ALT among control and treated rats. Indeed, previous reports have demonstrated that I3C and DIM attenuated liver inflammation in animals with ethanol- or staphylococcal enterotoxin B-induced acute liver injury [40,41]. Regarding chronic liver injury, Liu et al. showed that an 8-week DIM treatment alleviated hepatic steatosis and inflammation in mice with non-alcoholic steatohepatitis [42]. Compared to the aforementioned animal models with obvious hepatic inflammation, 4 weeks post CBDL, rats are characterized by jaundice, portal hypertension, liver fibrosis, and mild to moderate inflammation [43,44,45]. Therefore, the anti-inflammatory effects of I3C/DIM in 4 week-CBDL rats may not be comparable. However, experiments performed with a different time frame post CBDL to address the impacts of anti-inflammation on liver cirrhosis by I3C and DIM treatments are warranted in the future.

In summary, I3C and DIM ameliorated the development of portal hypertension and cirrhosis in CBDL rats and improved the cardiovascular function in non-cirrhotic control rats. The mitigation of severity of portosystemic collaterals and mesenteric angiogenesis is also noteworthy. However, cautions should be paid in translating the results of animal experiments. Although glucosinolates are present in relatively high concentrations in cruciferous vegetables, glucobrassicin, the glucosinolate precursor of I3C, makes up only about 8–12% of the total glucosinolates [46]. Furthermore, the amounts of I3C and DIM are variable and depend, in part, on the processing and preparation of foods. In fact, a nutritional company declared that it takes around 18 kg of broccoli to provide roughly 200 mg of I3C and 20 mg of DIM [47]. Based upon the current findings, the compounds might serve as pharmacological targets for the development of treatment agents despite supplying these doses via the diet.

## 4. Materials and Methods

### 4.1. Animal Model: Common Bile Duct Ligation (CBDL) Rats

Male Sprague-Dawley rats weighing 240–270 g at the time of surgery were used. Secondary biliary cirrhosis was induced via CBDL operation under ketamine anesthesia (100 mg/kg body weight, intramuscularly) [43]. Surgical control group received sham operation. Two ligatures, one of which near the junction of hepatic ducts and the other above the entrance of pancreatic duct, were tied then the CBD between two ligatures were cut apart. A high yield of secondary biliary cirrhosis was noted 4 weeks after the surgery. To avoid coagulation defects, CBDL rats received weekly vitamin K injections (50 μg/kg body weight, intramuscularly) [44,45].

### 4.2. Systemic and Splanchnic Hemodynamics and Biochemical Markers Measurement

The right femoral artery and superior mesenteric vein were cannulated using a PE-50 catheter connected to a Spectramed DTX transducer (Spectramed Inc., Oxnard, CA, USA). Continuous recordings of mean arterial pressure, heart rate and portal pressure were performed on a multi-channel recorder (model RS 3400, Gould Inc., Cupertino, CA, USA) [48,49]. The superior mesenteric artery flow was detected with a pulsed-Doppler flow transducer (T206 small animal blood flow meter, Transonic System Inc., Ithaca, NY, USA). Cardiac output was measured using a thermodilution method as previously described using a Columbus Instruments Cardiotherm 500-AC-R (Columbus Instruments International Co., Columbus, OH, USA) [50]. Cardiac index (mL/min/100 g body weight) was calculated as cardiac output per 100 g body weight. Stroke volume was calculated as cardiac output/heart rate (mL/beats). Systemic vascular resistance (mmHg/mL/min/100 g body weight) was calculated as mean arterial pressure/cardiac index. Superior mesenteric artery resistance (mmHg/mL/min/100 g body weight) was calculated as (mean arterial pressure–portal pressure)/superior mesenteric artery flow per 100 g body weight. Liver and kidney biochemical levels were measured: Alanine aminotransferase, total bilirubin, and creatinine.

### 4.3. Color Microsphere Method for Portosystemic Shunting Degree Analysis

The degree of portosystemic shunting was determined using the technique described by Chojkier and Groszmann whereas substituting color for radioactive microspheres according to the manufacturer’s instructions (Dye Track; Triton Technology, San Diego, CA, USA) [51]. Spillover between wavelengths was corrected using the matrix inversion technique. Portosystemic shunting was calculated as number of lung microspheres/(liver plus lung) microspheres. It has been shown that color microspheres provide results similar to those using radioactive microspheres [52].

### 4.4. Immunofluorescent Study for Mesenteric Vascular Density

Angiogenesis was quantified using CD31-labelled microvascular networks in rat mesenteric connective tissue windows according to a previous study with minor modifications [53]. From each rat, at least four mesenteric windows (wedge-shaped regions of connective tissue bordered by the intestinal wall and ileal blood vessel pairs) were dissected free and fixed in 100% MeOH (−20 °C for 30 min). Primary antibody mouse anti-rat CD31-biotin (1:200; AbD Serotec, Oxford, UK) and secondary antibody (CY2-conjugated streptavidin, 1:1000; Jackson ImmunoResearch, West Grove, PA, USA) were applied. At least four sets of data were obtained for each mesenteric window. Magnified immunofluorescent images (100×) were assessed using an upright fluorescent microscope (AX80, Olympus, Tokyo, Japan) with a charge-couple device (QICAM, High-performance IEEE 1394 FireWireTM Digital CCD Camera, Q IMAGING, BD, Canada) and thresholded by Image J software (available for download from the National Institutes of Health [54] as previously described [55].

### 4.5. Western Blotting for Protein Analysis

The concentrations of mesentery protein extracts were determined by the Bradford method [56]. Blots were incubated with the primary antibodies (anti-eNOS, anti-iNOS (1:1000; Millipore Corporation, Billerica, MA, USA); anti-phosphorylated eNOS (peNOS, 1:1000; Cell Signaling Technology, Danvers, MA, USA), anti-phosphorylated iNOS (piNOS, 1:1000; Abcam plc, Cambridge, UK); anti-NFκB p65 (1:200; Santa Cruz Biotechnology, Santa Cruz, CA, USA); anti-IκBα (1:1000; Abcam plc); anti-phosphorylated IκBα (pIκBα, 1:1000; Cell Signaling Technology); anti-Akt, -phosphorylated Akt Ser473 (pAkt) (1:1000 and 1:2000, respectively, Cell Signaling Technology); anti-Erk, -phosphorylated Erk (pErk) (1:3000, Millipore Corporation); anti-HIF-1α (1:200; Abcam plc); anti-Raf-1, -phosphoylated-Ser-338 Raf-1 (-pRaf-1), -VEGFR2, -phosphorylated-VEGFR2 (1:1000; Cell Signaling Technology); anti-VEGF, MMP-9 (1:1000, 90 min at room temperature, Santa Cruz Biotechnology); anti-beta-actin (1:5000; Cell Signaling Technology). The specific proteins were detected by enhanced chemiluminescence (Immobilon Western Chemiluminescent HRP Substrate, Merk Millipore Co., Billerica, MA, USA) and scanned with a computer-assisted video densitometer and digitalized system (BioSpectrum^®^ 600 Imaging System, Ultra-Violet Products Ltd., Upland, CA, USA). Then the signal intensity (integral volume) of the appropriate band was analyzed.

### 4.6. Determination of Plasma VEGF Concentration

Plasma levels of VEGF were measured using a commercially available enzyme-linked immunosorbent assay kit (R&D Systems Inc., Minneapolis, MN, USA) according to the manufacturer’s instructions.

### 4.7. Determination of Hepatic Fibrosis

The percentage of stained areas of paraffin-embedded liver sections were assessed using a Sirius red staining kit (Polysciences, Inc., Warrington, PA, USA) and Image J software [55].

### 4.8. Drugs

I3C was purchased from Sigma Chemical Co. (St. Louis, MO, USA), and DIM was purchased from Santa Cruz Biotechnology. All solutions were freshly prepared on the day of the experiment.

### 4.9. Statistical Analysis

Results are expressed as mean ± S.E.M. Statistical analyses were performed using an unpaired Student’s *t*-test for the comparisons of hemodynamic parameters between CBDL-vehicle and Sham-vehicle groups. ANOVA with the least significant difference test was applied for the comparisons among CBDL-vehicle, -DIM, -I3C groups, and among Sham-vehicle, -DIM, -I3C groups, respectively. Results were considered statistically significant at a two-tailed *p*-value of less than 0.05. The SPSS 21 statistical package for Windows (SPSS Inc., Chicago, IL, USA) was used.

### 4.10. Study Protocol

Rats were randomly allocated to receive I3C (20 mg/kg, intraperitoneal injection, which allowed for maximal systemic exposure according to the previous publication [57]), DIM (5 mg/kg/day, subcutaneous injection), or vehicle from the first day after CBDL and sham operations. Sham groups were used to monitor the potential influences of I3C and DIM on hemodynamics of normal subjects. On the 29th day after the surgery, the following were evaluated: Body weight, systemic and portal hemodynamics, plasma levels of liver and kidney biochemical parameters, portosystemic collateral shunting, mesenteric angiogenesis, plasma VEGF concentration, liver fibrosis, and mesenteric Western blot analysis for proangiogenic factor protein expressions. This study has been authorized by Taipei Veterans General Hospital Animal Committee with experimental approval (IACUC 2015-084, approved on 8th Jun., 2015). All animals received humane care according to the criteria outlined in the “Guide for the Care and Use of Laboratory Animals” published by the National Institutes of Health, the United States (NIH publication 86–23, revised 1985).

## Figures and Tables

**Table 1:** Body weight, hemodynamic parameters, and biochemical markers in CBDL (common bile duct ligation)-induced cirrhotic rats and sham rats with vehicle, DIM, or I3C treatment.

	CBDL-Vehicle *n* = 5	CBDL-DIM *n* = 7	CBDL–I3C *n* = 7	Sham-Vehicle *n* = 6	Sham-DIM *n* = 6	Sham-I3C *n* = 6
BW (g)	388.0 ± 20.2 ^#^	409.1 ± 4.2	408.0 ± 20.9	447.8 ± 11.6	432.7 ± 7.2	451.0 ± 10.8
MAP (mmHg)	120.7 ± 4.7 ^#^	108.9 ± 5.6	106.6 ± 8.9	145.96 ± 4.66	145.5 ± 5.0	150.0 ± 6.0
HR (beats/min)	363 ± 10	326 ± 11 *	361 ± 12	376 ± 19	353 ± 18	317 ± 27
PP (mmHg)	17.8 ± 1.2 ^###^	16.2 ± 0.6 *	16.1 ± 1.1 *	10.7 ± 0.3	9.5 ± 0.5	9.5 ± 0.5
CO (mL/min)	141.5 ± 5.5	120.7 ± 8.8	123.8 ± 6.0	131.3 ± 2.0	161.2 ± 11.6 *	140.1 ± 4.2
CI (mL/min/100 g)	36.4 ± 2.0 ^#^	29.4 ± 2.1	30.17 ± 3.1	29.4 ± 0.9	37.2 ± 2.3 **	31.1 ± 0.8
SV (mL/beats)	0.39 ± 0.02 ^###^	0.37 ± 0.02	0.34 ± 0.01	0.06 ± 0.00	0.07 ± 0.01 *	0.08 ± 0.00 *
SVR (mmHg/mL/min/100 g)	3.24 ± 0.56 ^##^	3.87 ± 0.27	3.70 ± 0.61	4.99 ± 0.24	3.97 ± 0.23 **	4.82 ± 0.13
SMA flow (mL/min/100 g)	5.9 ± 0.4 ^#^	6.2 ± 0.4	5.8 ± 0.8	4.7 ± 0.2	5.9 ± 0.6	5.6 ± 0.5
SMAR (mmHg/mL/min/100 g)	18.04 ± 2.15 ^##^	15.62 ± 1.64	17.54 ± 2.33	28.83 ± 1.20	24.31 ± 2.84	25.81 ± 1.58
ALT (U/L)	237.8 ± 40.0	206.1 ± 32.0	189.9 ± 29.3	45.8 ± 2.2	53.8 ± 2.6	48.3 ± 2.5
Total Bilirubin (mg/dL)	9.35 ± 0.64	8.64 ± 0.41	8.13 ± 0.82	0.02 ± 0.00	0.03 ± 0.00	0.01 ± 0.00
Creatinine (mg/dL)	0.52 ± 0.05	0.55 ± 0.03	0.57 ± 0.06	0.43 ± 0.03	0.42 ± 0.03	0.40 ± 0.01

BW: body weight; MAP: mean arterial pressure; HR: heart rate; PP: portal pressure; CO: cardiac output; CI: cardiac index; SV: stroke volume; SVR: systemic vascular resistance; SMA: superior mesenteric artery; SMAR: superior mesenteric artery resistance; ALT: alanine aminotransferase; * *p* < 0.05, ** *p* < 0.005, *** *p* < 0.001, DIM-or I3C-treated group vs. corresponding vehicle group analyzed with ANOVA; ^#^ *p* < 0.05, ^##^ *p* < 0.005, ^###^ *p* < 0.001, CBDL-vehicle vs. sham-vehicle group analyzed with *t*-test.
